# Modelling Clozapine Levels to Identify Safe Titration Targets and a Method for Precise Dose Adjustment

**DOI:** 10.1192/bjo.2024.210

**Published:** 2024-08-01

**Authors:** Zeryab Meyer, Gemma Simpson, Richard J Drake

**Affiliations:** 1 Division of Psychology & Mental Health, University of Manchester, Manchester, United Kingdom; 2 Northern Care Alliance NHS Foundation Trust, Greater Manchester, United Kingdom; 3 Pennine Care NHS Foundation Trust, Greater Manchester, United Kingdom; 4 Greater Manchester Mental Health Trust, Greater Manchester, United Kingdom

## Abstract

**Aims:**

A precision medicine approach to clozapine dosing aims to personalise it in two ways: i) during titration, and ii) for succeeding dose adjustments. This requires valid models of dose/concentration relationships, but cross-sectional models suffer from population-level artefacts and individual problems due to poor adherence or stopping smoking. Longitudinal data sets from two mental health trusts allowed poor adherence and smoking cessation to be identified. We then modelled dose/concentration relationships to construct personalised targets for i) and ii).

**Methods:**

Demographics and co-prescribed medication were recorded for 137 patients from Greater Manchester Mental Health (GMMH) Trust who had two or more successive plasma levels and doses from 2016–2018. 412 patients from Pennine Care Foundation Trust (PCFT) who had successive plasma levels and doses from 2009–2023 were also recorded. In each sample, adherent patients (88 from GMMH and 371 from PCFT) were identified after excluding: > two-fold variation between blood samples in clozapine/norclozapine ratios, > two-fold variation in dose/concentration ratios, a clozapine/norclozapine ratio > 3, or a dose/concentration ratio > two standard deviations from the sample mean. Those whose smoking status (smoker vs non-smoker) changed between samples were excluded.

To identify i) titration targets, we used raw data in first samples (checked with logistic regression) to identify dose thresholds which produced most levels above 0.35 μg/ml (therapeutic) and no levels greater than 1 μg/ml (toxic). To model ii) effective dose adjustment, we used the equation Dt = Dc(Ct/Cc) to identify the most effective dose for the second samples. Dt was target dose, Dc current dose, Ct target level (0.45 μg/ml), and Cc current level.

**Results:**

First sample dose/concentration ratio in adherent patients correlated r > 0.75 with second samples’ dose/concentration. >84% of plasma levels were within 20% of the mean across both samples.

- The GMMH dataset titration targets were 325 mg, 300 mg, 225 mg, and 175 mg daily for male smokers, female smokers, male non-smokers, and female non-smokers, respectively. In PCFT, data suggested corresponding targets of 375 mg, 325 mg, 225 mg and 175 mg. Targets avoided toxicity and gave therapeutic levels in > 50% of cases.
- Target dose, ascertained using the equation, and actual second dose were compared: in adherent cases, toxicity only occurred when actual doses were 1.5-fold greater than target dose, and above target all plasma levels exceeded 0.35 μg/ml in GMMH. PCFT data appeared similar.

**Conclusion:**

Relatively safe and effective titration targets for smokers and non-smokers from both sexes were identified. A simple equation would improve precision and effectiveness of dose adjustment thereafter.

